# Cinnamon nanoemulsion mitigates acetamiprid-induced hepatic and renal toxicity in rats: biochemical, histopathological, immunohistochemical, and molecular docking analysis

**DOI:** 10.1186/s12917-024-04084-x

**Published:** 2024-06-12

**Authors:** Ahmed A. A. Aioub, Sameh A. Abdelnour, Ahmed S. Hashem, Mohamed Maher, Sarah I. Z. Abdel-Wahab, Lamya Ahmed Alkeridis, Mustafa Shukry, Samy M. Sayed, Ahmed E. A. Elsobki

**Affiliations:** 1 Plant Protection Department, Faculty of Agriculture, Zagazig University, Zagazig, 44511 Egypt; 2 Animal Production Department, Faculty of Agriculture, Zagazig University, Zagazig, 44511 Egypt; 3 Stored Product Pests Research Department, Plant Protection Research Institute, Agricultural Research Center, Sakha, Kafr El-Sheikh, 33717 Egypt; 4 Department of Biochemistry, Faculty of Agriculture, Zagazig University, Zagazig, 44511 Egypt; 5 Department of Biology, College of Science, Princess Nourah Bint Abdulrahman University, P.O. Box 84428, Riyadh, 11671 Saudi Arabia; 6 Physiology Department, Faculty of Veterinary Medicine, kafrelsheikh University, kafrelsheikh, 33516 Egypt; 7 Department of Economic Entomology and Pesticides, Faculty of Agriculture, Cairo University, Giza, 12613 Egypt; 8 Department of Science and Technology, University College-Ranyah, Taif University, B.O. Box 11099, Taif, 21944 Saudi Arabia

**Keywords:** Acetamiprid, Cinnamon nanoemulsions, Biochemical changes, Histological and immunobiological analysis, In silico analysis

## Abstract

Acetamiprid (ACDP) is a widely used neonicotinoid insecticide that is popular for its efficacy in controlling fleas in domestic settings and for pets. Our study aims to offer a comprehensive examination of the toxicological impacts of ACDP and the prophylactic effects of cinnamon nanoemulsions (CMNEs) on the pathological, immunohistochemical, and hematological analyses induced by taking ACDP twice a ‎week for 28 days. Forty healthy rats were divided into four groups (*n* = 10) at random; the first group served as control rats; the second received CMNEs (2 mg/Kg body weight); the third group received acetamiprid (ACDP group; 21.7 mg/Kg body weight), and the fourth group was given both ACDP and CMNEs by oral gavage. Following the study period, tissue and blood samples were extracted and prepared for analysis. According to a GC-MS analysis, CMNEs had several bioactive ingredients that protected the liver from oxidative stress by upregulating antioxidant and anti-inflammatory agents. Our findings demonstrated that whereas ACDP treatment considerably boosted white blood cells (WBCs) and lymphocytes, it significantly lowered body weight gain (BWG), red blood cells (RBCs), hemoglobin (Hb), hematocrit (HCT), and platelets (PLT). ACDP notably reduced antioxidant enzyme activities: superoxide dismutase (SOD), glutathione peroxidase (GPx), and catalase (CAT) and elevated hydrogen peroxide and malondialdehyde levels compared with other groups. ACDP remarkably raised alanine aminotransferase (ALT), aspartate amino transaminase (AST), and alkaline phosphatase (ALP) levels.

Moreover, the histopathological and immunohistochemistry assays discovered a severe toxic effect on the liver and kidney following ACDP delivery. Furthermore, cyclooxygenase 2 (COX-2) + immunoexpression was enhanced after treatment with CMNEs. All of the parameters above were returned to nearly normal levels by the coadministration of CMNEs. The molecular docking of cinnamaldehyde with COX-2 also confirmed the protective potential of CMNEs against ACDP toxicity. Our findings highlighted that the coadministration of CMNEs along with ACDP diminished its toxicity by cutting down oxidative stress and enhancing antioxidant capacity, demonstrating the effectiveness of CMNEs in lessening ACDP toxicity.

**Supplementary Information:**

The online version contains supplementary material available at 10.1186/s12917-024-04084-x.

## Introduction

Pesticides stand out as one of the most pervasive types of pollutants worldwide but come with significant drawbacks, primarily their toxicity [1]. Poorly executed spraying techniques, whether from misuse, overuse, or intentional abuse, can result in considerable adverse effects on crop production while also posing risks to the environment and human health [2] and inducing potential toxicity to humans [3] as well as non-target organisms [4]. Therefore, long-term exposure can harm human life and interfere with the operation of diverse body organs [5, 6]. Consequently, pesticide side effects have drawn attention from across the world.

Neonicotinoids are a class of insecticides that have recently gained popularity in agricultural pest control [7]. One of the neonicotinoid pesticides, acetamiprid (ACPD), operates on insects’ acetylcholine (nACh) receptors and has rapidly gained attraction in the international market [8]. ACPD is water soluble, easily absorbed by plants through their leaves or roots, distributed throughout plant tissues, and prone to leaching into waterways and soil wastes [9]. Therefore, it can accumulate in the human body or non-target organisms [10]. After inhaling ACPD, patients experience headaches, dizziness, nausea, vomiting, and other symptoms [11], and it reaches the liver, kidney, adrenal glands, and thyroid [12]. Various studies have proved that exposure to ACPD induced hepatotoxicity [11, 13], neurotoxicity [14], cytotoxicity, and genotoxicity [15].

Multifarious in vivo and in vitro studies have claimed that ACPD evoked a disproportion between the body’s antioxidant defense system and oxidants, generating oxidative stress [15]. In addition to impairing the cellular functioning of diversified organs and degrading cellular macromolecules like proteins and lipids, oxidative stress also lowers antioxidant levels. It heightens lipid peroxidation, DNA damage, and genotoxicity [16]. Oxidative stress production has been discovered to be crucial in liver damage [11] and kidney injury [17] caused by ACMP. Another finding from a recent study was the dysregulation and abnormality in amino acid and fatty acid metabolism, which changed the biochemistry of lipids and may have contributed to the development of oxidative stress in liver tissue [13]. Moreover, the oxidative stress produced by ACPD leads to severe oxidative damage to hepatic cells, which results in hepatic cell death and malfunction. These data indicate that oxidative stress will always play a role in ACMP-induced liver damage.

Cinnamon (*Cinnamomum verum*) is a traditional medicinal herb used to treat diabetes, inflammatory diseases, and cancers [16]. Cinnamaldehyde, cinnamic acid, and other distinct chemicals present in cinnamon, together with essential oils, exhibit a range of biological activities, such as antioxidant [18], anti-inflammatory [19], and antitumor properties [20]. Moreover, cinnamon exhibits hepatoprotective and nephroprotective effects [21]. Furthermore, cinnamon has hepatoprotective activity against alcohol and carbon tetrachloride-induced hepatic injury [22]. Likewise, Sakr et al. [23] pinpointed that cinnamon was protective in kidney injury induced by cypermethrin.

With its vast array of potential applications, nanotechnology has experienced tremendous progress [24]. A type of emulsion known as nanoemulsions has droplet sizes ranging from 20 to 500 nm [25]. Nanoemulsions are employed in various biological applications because of their unique features, such as strong stability and controllable rheology [26]. Some studies have proven that cinnamon was utilized to lessen the toxicity and oxidative stress caused by pesticides in living things [27]. However, no study has been published till now about using cinnamon in CMNEs form as an ameliorating effect against ACPD in rats.

Consequently, we hypothesized that CMNEs might enhance antioxidant capacity and reduce proinflammatory mediators in male rats, thereby counteracting the harmful effects of ACPD-induced toxicity. This study aimed to examine the ameliorative function of CMNEs against the deleterious effects of ACPD on liver and renal tissues, as well as the combined and independent effects of ACPD and CMNEs on body weight growth in test rats. Furthermore, hematological parameters, histopathological alterations, oxidative stress levels, and antioxidant enzyme levels were assessed in both liver and kidney tissues. Additionally, the liver and kidney tissues were subjected to immunohistochemical analysis to evaluate the expression of COX-2 under the influence of ACPD stress. Moreover, molecular docking was performed to investigate the interaction between the primary component of CMNEs and COX-2, with cinnamaldehyde acting as the ligand and COX-2 as the receptor.

## Materials and methods

### Chemicals and reagents

Acetamiprid (ACPD, 97%) was obtained from Sigma, China. ALT, AST, ALP, SOD, CAT, malondialdehyde (MDA), hydrogen peroxide (H_2_O_2_), and GPx kits were obtained from Biodiagnostic Company (Dokki, Giza, Egypt).

### Nanoemulsion preparation and characterization

The nanoemulsion (NE) containing Cinnamomum verum essential oil (EO) was synthesized at the Agricultural Research Center in Sakha, Kafr El-Sheikh (coordinates: 66.7613° N, 124.1238° E) using the method outlined by Hamouda et al. Hamouda et al. [28], with slight modifications as described by Hashem et al. Hashem et al. [29]. Briefly, Thickened O/W nanoemulsions were made using Tween 80 (3%, v/v), ethanol (3%, v/v), and C. verum essential oil (14%, v/v of the total coarse emulsion), which accounted for 20% (v/v) of the overall emulsion. After mixing, the oil phase components were stored at 86 °C for one hour. After that, they were combined with 80% water, left for three minutes, and then centrifuged at 10,000 ×g. The cinnamon oil nanoemulsion was kept in dark vials at room temperature until more analysis.

Concerning the characterization of the cinnamon oil nanoemulsion, the dynamic laser light-scattering method (DLS) was utilized to ascertain the droplet size distribution (analysis by volume). Zeta potential, viscosity, conductivity, and polydispersity index (PDI) were investigated by photon correlation spectroscopy (Malvern Zetasizer Nano-zs90, Malvern Instruments Ltd., Enigma Business Park, Grovewood Road, Malvern, Worcestershire, WR14 1XZ, UK). The morphological examination of the cinnamon oil nanoemulsion was performed by adopting a JEOL JEM-2100 transmission electron microscope (TEM) operating at 120 kV. The samples were placed on copper grids and allowed to dry at room temperature. Then, the TEM was inspected without staining.

### Gas chromatography-mass spectrometry (GC–MS) analysis

The phytoconstituents of CMNEs were analyzed by a TRACE™ 1310 gas chromatography equipped with Fisher Trace ISQ mass spectrometer (GC-MS) column (SLB™-5ms 30 m × 0.25 mm × 0.25 μm, Sigma-Aldrich, St. Louis, MO, USA). A helium (purity 6.0, Westfalen AG, Münster, Germany) carrier gas flow rate of 1.5 mL min^−1^ was utilized with a sample injection volume of 1 µL and a split of 1:5 at an injection temperature of 250 °C. Regarding GC settings, the oven program was started at 40 °C for 1 min and increased at 3 K min^−1^ to 60 °C and then at 30 K min^−1^ until 280 °C was reached; the final temperature was kept constant for 8 min [30]. Conversely, the MS operating settings were 250 ◦C for the injector and MS transfer line, 250 ◦C for the ion source, 70 eV for the ionization energy, and a mass scan range of 50–500 amu for the complete ion scan mode (scan time: 0.2 s) [31]. The identification of compounds in both samples involved a comparison of their linear retention indices (RI), retention times (RT), and mass spectra with those obtained from authentic samples (gained from the Sigma-Aldrich Group) and/or the NIST/NBS, Wiley libraries, and relevant literature. Subsequently, individual compounds in both samples were determined by analyzing the peak area on the GC chromatogram [32].

### Animal and experimental design

Forty adult Sprague Dawley rats, aged between 13 and 15 weeks and weighing an average of 160 ± 20 g, were sourced from the Laboratory Animal Housing Unit of the Faculty of Veterinary Medicine at Zagazig University in Egypt. These rats were housed in stainless-steel cages under a 12-hour light/dark cycle in a well-ventilated room, ensuring optimal conditions for their well-being. Throughout the study, the rats had ad libitum access to food and water. Animal care and experimental procedures complied with protocols approved by the Zagazig University of Egypt’s Faculty of Agriculture Committee (ZU-IACUC/2/F/132/2023), ensuring ethical standards and regulatory requirements were met.

Before the tests began, the experimental animals spent a week becoming used to the lab environment. Forty rats were divided into four experimental groups at random (10 rats /group); Group I, the control group, was administered orally daily with normal saline; Group II received CMNEs orally daily (2 mg/kg bwt) only [33]; Group III was given ACPD orally twice a week at a dosage of 1/10 LD_50_ (21.7 mg/kg bwt) [11]; Group IV received CMNEs after 30 min of ACPD administration orally. z. Rats were examined, and any toxicity-related clinical symptoms were recorded daily. The body weight gain was calculated using the formula: weight gain = [(final body weight − initial body weight)/initial body weight] ×100.

### Sampling

The animals were weighed the night after their 28-day treatment period and then starved. Animals had a fasting period of about 12 to 18 h before anesthesia. The experimental animals were anesthetized by isoflurane inhalation (Anesthesia was initiated by placing the rats inside an anesthesia induction chamber (measuring 25 × 25 × 14 cm) where they were exposed to 4% isoflurane (Forane; Abbott Japan Co., Ltd., Tokyo, Japan) following [34] and decapitated to obtain blood samples. The samples were collected in tubes using 10% EDTA as an anticoagulant for hematological analysis. A portion of the blood samples was stored in tubes and given 30 min to coagulate at room temperature, then centrifuged at 3,000 rpm for 20 min. After that, the serum was kept at -20 °C for additional testing (liver enzymes and kidney function markers). Subsequently, the kidney and liver tissues were collected, and part was homogenized at a 10% (w/v) concentration in potassium phosphate buffer (pH 7.4) using a tissue homogenizer. Following homogenization, the mixture was centrifuged at 3000 g for 10 min at 4 °C. The resultant supernatant was then stored at -20 °C; another part was used for histological and immunohistopathological analyses; the livers and kidneys were removed, saline-washed, and preserved in neutral buffered formalin (10%).

### Hematological parameters

The obtained blood samples were examined to ascertain the erythrogram and leukogram profiles, among other hematological characteristics. The erythrogram profile involving (RBC, 10^12^/L), (Hb, g/L), (HCT, %), mean corpuscular volume (MCV, fL), mean corpuscular hemoglobin (MCH, pg), mean corpuscular hemoglobin concentrations (MCHC, g/dL), (PLT, 10^9^/L), mean cell volume (MCV, fL), and the leukogram prole such as (WBC, 10^9^/L), lymphocyte percentage, and complete blood counts were estimated via an automated blood cell analyzer (Hemascreen18, Hospitex Diagnostics, Sesto Fiorentino, Italy).

### Antioxidant profile

SOD activity was measured using a kit SOD (cat.no. SD 2521) from Biodiagnostic, Cairo, Egypt, and a technique by Nishikimi et al. [35] at 440 nm. CAT activity was measured by Aebi [36] using a kit (cat. no. CA-2517) obtained from Biodiagnostic, Cairo, Egypt, and expressed in units per gram of tissue (U/g) at 510 nm. GPx activity was evaluated using spectrophotometry by a prior investigation by Paglia and Valentine [37]. GPx activity was determined by oxidizing NADPH and GSH with glutathione reductase, detecting the drop in absorbance at 340 nm, and expressing the result in units/mg protein using GPX (cat. no. 2524, Biodiagnostic). Protein levels in kidney homogenates were quantified using the method outlined by Lowry et al. [38] with bovine serum albumin serving as the standard. see the supplementary file.

### Oxidative stress measurements

The MDA was expressed as nmol/mg protein and measured at 532 nm using a kit from Biodiagnostic (cat. no. MD-2529), Cairo, Egypt. The kit was developed Ohkawa et al. [39]. H_2_O_2_ was measured at 610 nm using the methodology proposed by Pick and Keisari [40] utilizing a kit (cat no. MBS841818; MyBioSource, San Diego, USA).

### Liver and kidney function

According to earlier research by Reitman and Frankel [41], The measurement of serum concentrations of alanine aminotransferase (ALT) and aspartate aminotransferase (AST) was evaluated calorimetrically using kits (cat. no. AT-1034; Bio-diagnostic). Meanwhile, the levels of ALP were estimated following the methodology of Ellis et al. [42]. Reagent kits from Biomed Diagnostics (Egypt) were utilized to determine serum creatinine, urea, and uric acid levels.

### Histopathological examination

The liver and kidney samples were cleaned with xylene, embedded in paraffin, and stored in a 10% formalin solution using an automated tissue processor. After that, 5 μm thick slices were created using a rotating microtome and stained with hematoxylin and eosin [43]. Subsequently, five slices of each test rat’s liver and kidney that had been stained were examined under a microscope at different magnifications to evaluate qualitative histological variations and perform histomorphometric analysis. For each investigated organ, the histomorphological alterations in five fields per section were used to grade the histopathological abnormalities in the hepatic and renal tissues. ImageJ was used to quantify changes observed under a microscope before statistical analysis initially.

### Immunohistochemical study

To perform immunohistochemical analysis, the avidin-biotin-peroxidase complex procedure, as outlined by Hsu et al. [44], was used to stain COX-2 antigens in the hepatic and renal tissues using rabbit monoclonal anti-COX-2 antibody (ab15191) (Abcam, United Kingdom) and 3,30-diaminobenzidine chromogen (DAB). In addition, To confirm whether the IHC analysis was selective and could eliminate nonspecific responses and false-positive results, the negative controls were treated with phosphate buffer saline rather than primary antibodies [45]. The DAB density did not correlate with the epitope concentration, and the majority of hepatic or renal cells were immune-positive to a variable degree for both indicators. Thus, the fractions of DAB brown spots to the total image regions were calculated to get a quantitative evaluation of the COX-2 immunodepression. Five fixed-size microscopic photographs of organisms or animals were taken using the open-source ImageJ program version 1.41 at the same magnification (×40) and exposure time.

### Target proteins and docking studies

The Protein Data Bank (PDB) website was operated to obtain the crystal structures of COX-2 (PDB ID: 6COX). According to GC-MS analysis, cinnamaldehyde was the highest concentration in the CMNEs. Cinnamaldehyde was selected as the ligand of COX-2 protein modeling. Then, cinnamaldehyde was acquired from PubChem and ChemSpider databases and was prepared by Molecular Operating Environment (MOE) 2014.13 software (Chemical Computing Group Inc, Montreal, Quebec, Canada) [46]. The ligands were decreased by using the CHARMM 99 force field before initiating the docking process. Ramachandran’s plot (PROCHECK analysis) was used to evaluate and validate these models. After that, bonds were added, duplicates were eliminated, and three-dimensional (3D) structures were built. After all default parameters were set and the least energy structures were obtained, the ligands were made flexible and manually inserted into the catalytic site cavity of the enzyme model. The binding energy was examined using a full-force field, and the ligand and protein’s affinity was evaluated using scoring systems that produced free-binding interaction energies based on molecular force field terms. The optimal ligand interaction was examined and assessed following docking using scoring methods and root-mean-square deviation (RMSD) calculations [47].

### Statistical analysis

GraphPad Prism program v.8 (GraphPad Software Inc., La Jolla, CA, United States) was used for data analysis. For multiple comparisons between the groups, we ran a one-way analysis of variance and then handled Tukey’s post-hoc test; a statistically significant difference was defined as *p* < 0.05.

## Results

### Characterization of nanoemulsion

**Fig. 1:**
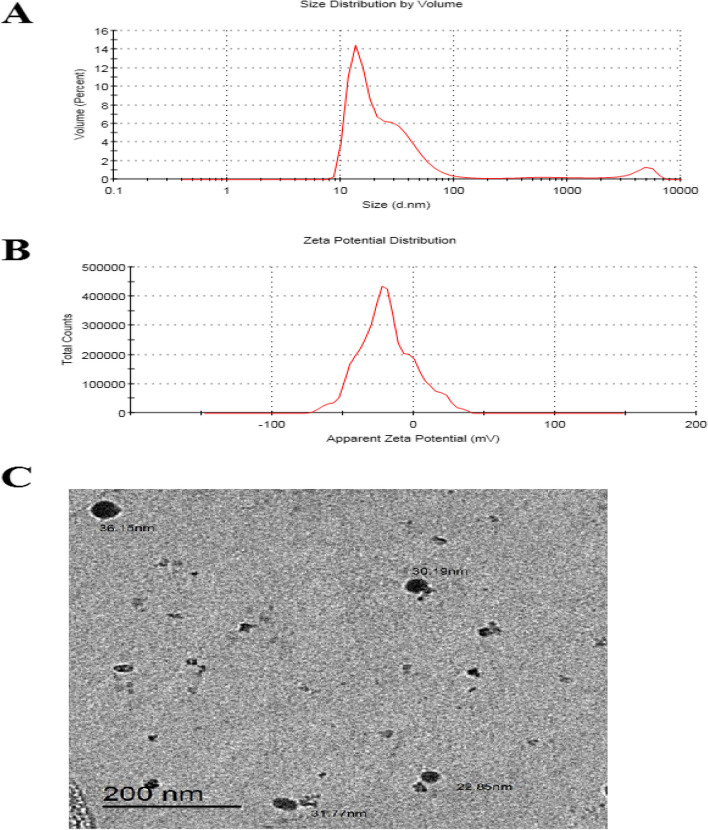
Characterization of cinnamon oil nanoemulsion. **A** Particle size distribution observed in Dynamic Light Scattering (DLS) instrument, (**B**) Surface net negative charge by Zeta potential, and (**C**) TEM image observe morphology of spherical nanoemulsion

The preparation of *C. verum* EO nanoemulsion involved high-pressure homogenization, resulting in a mean droplet size ranging from 100 nm to 200 nm (expressed as z-diameter), with the majority being 157 nm in size (Fig. 1A). The polydispersity index, which typically ranged between 0.23 and 0.25, reflected a highly uniform distribution of droplet sizes with minimal variation (Fig. 1B). Furthermore, zeta potential (mV), conductivity (mS/cm), and viscosity (cP) of *C. verum* EO-based NE were as follows: −24.2 ± 4.39, 0.028, and 0.887, respectively. Moreover, TEM showed that the particle size ranged from 22 to 37 nm and the droplets were spherical (Fig. 1C).

### Identification of CMNEs by GC–MS

The GC-MS chromatogram of CMNEs recorded 32 volatile organic compounds (VOCs). According to their retention duration, peak area, height, molecular weight, molecular formula, and mass spectrum records of the known compounds kept by the National Institute of Standards and Technology (NIST) library, each compound identified the phytocompounds (Table 1). The main components of CMNEs are cinnamaldehyde (37.27%), benzyl alcohol (17.59%), bicyclo[2.2.1]heptan-2-one,1,7,7-trimethyl-(1 S) (8.83%), benzene ethanol (8.54%), and trans-13-octadecenoic acid, methyl ester (7.71%).

**Table 1:** GC-MS spectral analysis of cinnamon nanoemulsion (CMNEs)

**No.**	**RT**^a^	**Conc. (%)**	**Compound**	**Molecular formula**
1	5.23	17.59	Benzyl alcohol	C_7_H_8_O
2	6.05	0.17	2-Naphthol,1,2,3,4,4a,5,6,7-octahydro-4a-methyl	C_11_H_18_O
3	6.36	0.17	9,12,15-OCTADECATRIENOIC ACID, METHYL ESTER	C_19_H_32_O_2_
4	6.80	8.54	BENZENEETHANOL	C_8_H_10_O
5	7.37	8.83	Bicyclo[2.2.1]heptan-2-one,1,7,7-trimethyl-, (1 S)-	C_10_H_16_O
6	8.88	0.51	Estragole	C_10_H_12_O
7	10.27	37.27	Cinnamaldehyde, (E)-	C_9_H_8_O
8	10.98	0.38	Anethole	C_10_H_12_O
9	12.69	0.36	Phenol, 2-methoxy-4-(1-propenyl)-	C_10_H_12_O_2_
10	13.19	1.59	2-PROPENOIC ACID, 3-PHENYL-,METHYL ESTER	C_10_H_10_O_2_
11	15.10	0.55	cis-à-Bergamotene	C_15_H_24_
12	16.79	0.25	á-copaene	C_15_H_24_
13	19.52	0.50	tau.-Cadinol	C15H26O
14	21.52	0.30	Benzene, [[(1-ethenyl-1,5-dimethyl-4-hexenyl) oxy]methyl]-	C_17_H_24_O
15	23.96	0.61	2,6,10,14-Hexadecatetraenoic acid, 3,7,11,15-tetramethyl-, methyl ester, (E, E,E)-	C_21_H_34_O_2_
16	24.20	0.23	Ethanol, 2-(9-octadecenyloxy)-, (Z)-	C_20_H_40_O_2_
17	25.19	0.23	1-(2-NITROCYCLOPENTYL)-3-PHENYL-2-PROPEN-1-OL	C_14_H_17_NO_3_
18	25.66	0.40	HEXADECANOIC ACID, METHYL ESTER	C_17_H_34_O_2_
19	26.44	1.06	HEXADECANOIC ACID	C_16_H_32_O_2_
21	28.24	0.29	Bicyclo[3.1.1]hept-3-ene-spiro-2,4'-(1',3'-dioxane), 7,7-dimethyl	C_12_H_18_O_2_
22	28.67	4.22	Methyl 9-cis,11-trans-octadecadienoate	C_19_H_34_O_2_
23	28.85	7.71	trans-13-Octadecenoic acid, methyl ester	C_19_H_36_O_2_
24	29.41	1.00	Methyl stearate	C_19_H_38_O_2_
25	29.60	1.19	[5,9-Dimethyl-1-(3-phenyl-oxiran-2-y l)-deca-4,8-dienylidene]-(2-phenyl-aziridin-1-yl)-amine	C_28_H_34_N_2_O
26	30.81	0.31	RETINOL	C_19_H_30_O_2_
27	35.84	2.01	Diisooctyl phthalate	C_24_H_38_O_4_
28	41.03	0.18	4 H-1-BENZOPYRAN-4-ONE, 2-(3,4-DIMETHOXYPHENYL)-3,5- IHYDROXY-7-METHOXY	C_18_H_16_O_7_
29	42.00	0.22	Ethyl iso-allocholate	C_26_H_44_O_5_
30	42.32	0.70	9,12-OCTADECADIENOIC ACID (Z,Z)-,2,3-BIS[(TRIMETHYLSILYL)OXY]PROPYL ESTER	C_27_H_54_O_4_Si_2_
31	43.08	1.67	DOTRIACONTANE	C_32_H_66_
32	44.25	0.19	1-Heptatriacotanol	C_37_H_76_O
33	44.76	0.76	Cyclobarbital	C_12_H_16_N_2_O_3_
^a^*RT* Retention time				

### Changes in body weight gain

**Fig. 2:**
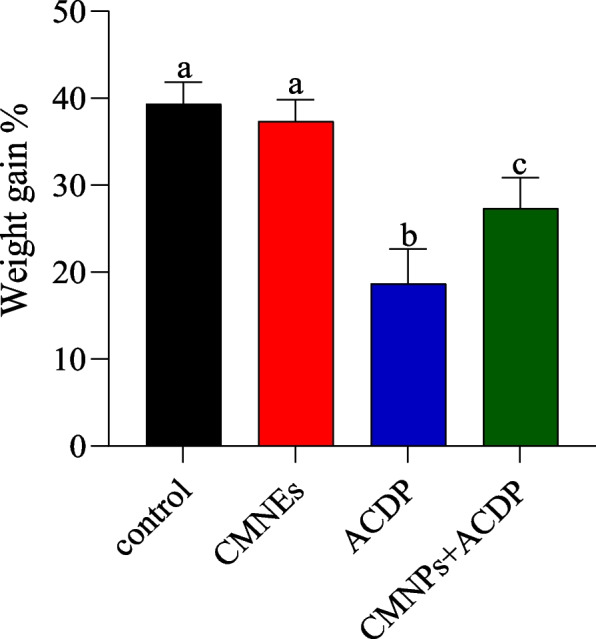
Effect of cinnamon nanoemulsion (CMNEs; G II) and acetamiprid (ACPD; G III) alone and together (G IV) for 28 days on rat’s body weight gain (%) compared with control (G I). The different letters represent the statistically significant differences at (*p* < 0.05) for comparison between all groups, followed by Tukey’s post hoc test

According to Fig. 2, ACDP exposure significantly lowered body weight gain (BWG) (*p* < 0.05) concerning the other treated groups. Control and CMNEs groups had similar weight gains, considerably higher than the ACDP group, which had the most minor gain; the CMNPs + ACDP group showed improved weight gain compared to ACDP alone but did not reach the levels of the control or CMNEs groups, suggesting some mitigation by CMNPs of ACDP’s adverse effects on weight.

### Hematological parameters changes by ACDP and CMNEs

Table 2 exhibited a substantial decrease in RBCs, HCT PLT, and Hb in the animals exposed to ACDP alone or co-administrated with CMNEs compared to the control and CMNEs groups. (*p* < 0.05). On the other hand, rats that received ACDP alone or with CMNEs displayed considerably higher WBC and lymphocyte levels than the control group (*p* < 0.05). Still, the CMNPs + ACDP showed a significant decrease in the total leukocytic count, accompanied by nonsignificant reductions in the lymphocytes of the ACDP-treated rats. In general, the control and CMNEs groups demonstrated improved hematological parameters.

**Table 2:** Hematological parameters and blood indices values of control and experimental rat

**Parameters**	**Control**	**CMNEs**	**ACDP**	**CMNPs + ACDP**	***P*-value**
**RBCs (**10^12^/L)	2.60^a^	2.39^b^	1.91^c^	1.94^c^	0.0002
**Platelet (PLT) (**10^9^/L)	77.67^a^	81.86^a^	50.33^b^	48.14^b^	0.0003
**Hemoglobin (Hb) (**g/L)	8.71^a^	8.62^a^	6.80^b^	6.98^b^	0.0002
**Hematocrit (HCT) %**	36.12^a^	35.09^a^	29.97^b^	29.86^b^	0.0001
**WBCs (**10^9^/L)	6.97^c^	6.88^c^	10.73^a^	8.65^b^	0.0002
**Lymphocytes (%)**	78.24^b^	78.70^b^	81.95^a^	81.38^a^	0.0124

Different letters represent significant differences (Tukey’s posthoc test significant difference test among all groups)

*RBC* red blood cell, *WBC* white blood cell

### Biochemical evaluation

**Fig. 3:**
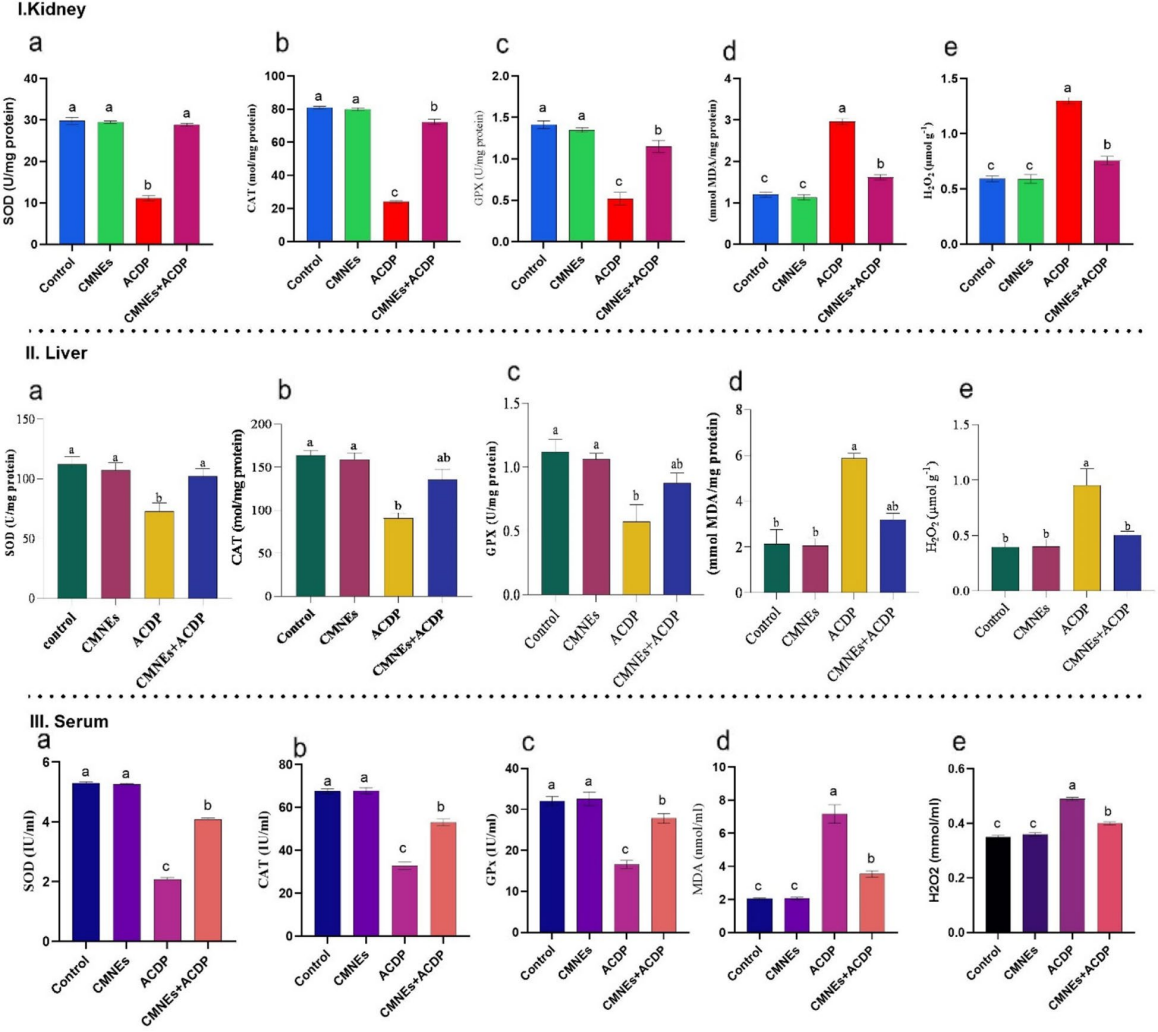
Effects of cinnamon nanoemulsion (CMNEs; G II) and acetamiprid (ACPD; G III) alone and together (G IV) on oxidative stress and antioxidant markers on the kidney (I), liver(II) and serum (III) of rats (*n* = 10) compared with control (G I). SOD = superoxide dismutase (**a**); CAT = catalase (**b**); GPx = glutathione peroxides (**c**); H_2_O_2_ = hydrogen peroxide (**e**); MDA = malondialdehyde (**d**); ALT = alanine aminotransferase (**f**); AST = aspartate amino transaminase (**g**); ALP = alkaline phosphatase (**h**). The letters represent the statistically significant differences at (*p* < 0.05) for comparison between all groups, followed by Tukey’s post hoc test

Figure 3I and II depicted statistically noteworthy variations between the levels of oxidative stress biomarkers (MDA and H_2_O_2_) and antioxidant enzymes (SOD, CAT, and GPx) in the hepatic, renal, and serum of treated and controlled rats. The ACDP-treated rats displayed significantly decreased SOD, CAT, and GPx activity in contrast to the dramatically increased MDA and H_2_O_2_ levels. In the CMNEs + ACDP-treated rat, the GPx, SOD, and CAT levels were lessened, but MDA and H_2_O_2_ were elevated compared to the control.

### Hepatic and renal function biomarkers

**Fig. 4:**
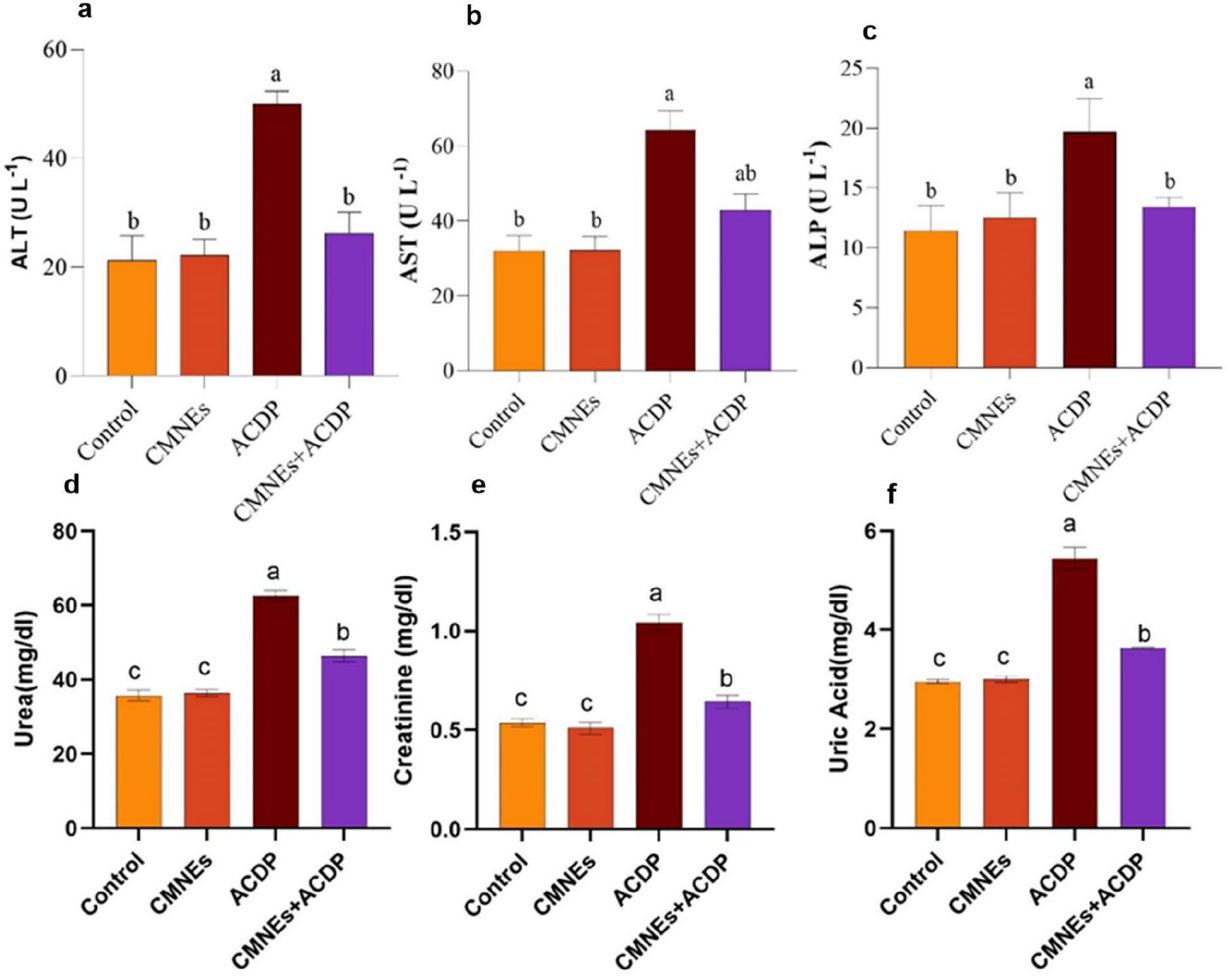
Effects of cinnamon nanoemulsion (CMNEs; G II) and acetamiprid (ACPD; G III) alone and together (G IV) on hepatic and renal function markers (*n* = 10) compared with control (G I). ALT= (**a**); AST (**b**); ALP (**c**); Urea (**d**; Creatinine (**e**); Uric Acid (**f**); The different letters represent the statistically significant differences at (*p* < 0.05) for comparison between all groups followed by Tukey’s post hoc test

ALP, AST, and ALT activity levels in addition to urea, uric acid, and creatinine in ACDP-treated rats, were noticeably higher than in control (*p* < 0.05). Additionally, rats treated with ACDP + CMNEs showed notably reduced levels of ALT, ALP, AST, urea, uric acid, and creatinine activity than those in the control and ACDP-only groups (*p* < 0.05) (Fig. 4).

### Histopathological findings

**Fig. 5:**
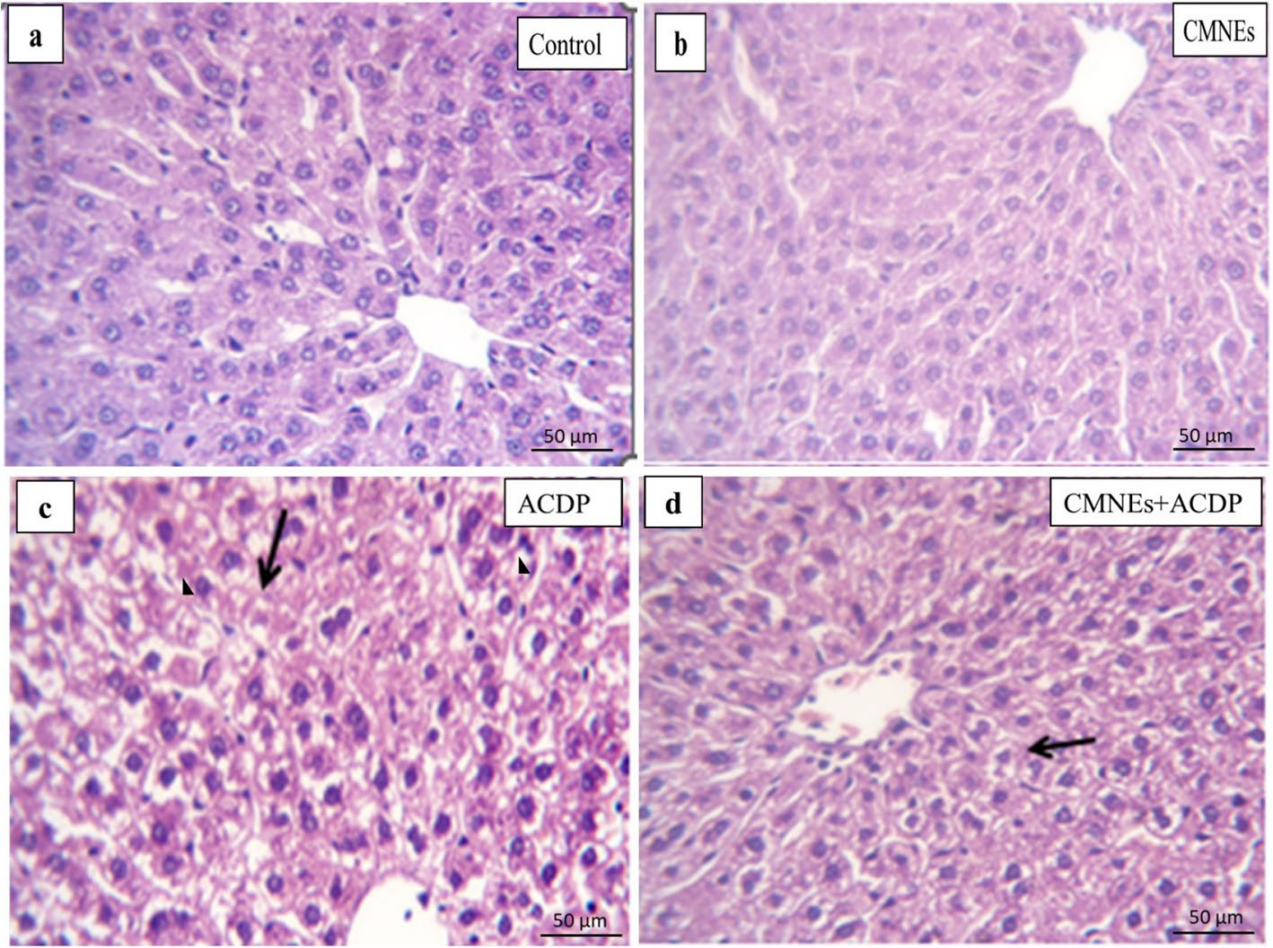
Photomicrograph of H&E-stained sections from the liver showing degenerative changes, necrotic changes, congested blood vessels, and round cells infiltration. **a** normal central vein and hepatic parenchyma. **b** normal cytoarchitectures of hepatic parenchyma. **c** severe degenerated and necrotic hepatocytes (arrow) with congested hepatic blood vessels (arrowhead) in the rat’s group treated with ACDP (arrow). **d** normal hepatic cords with degenerative changes within some hepatocytes (arrow)

**Fig. 6:**
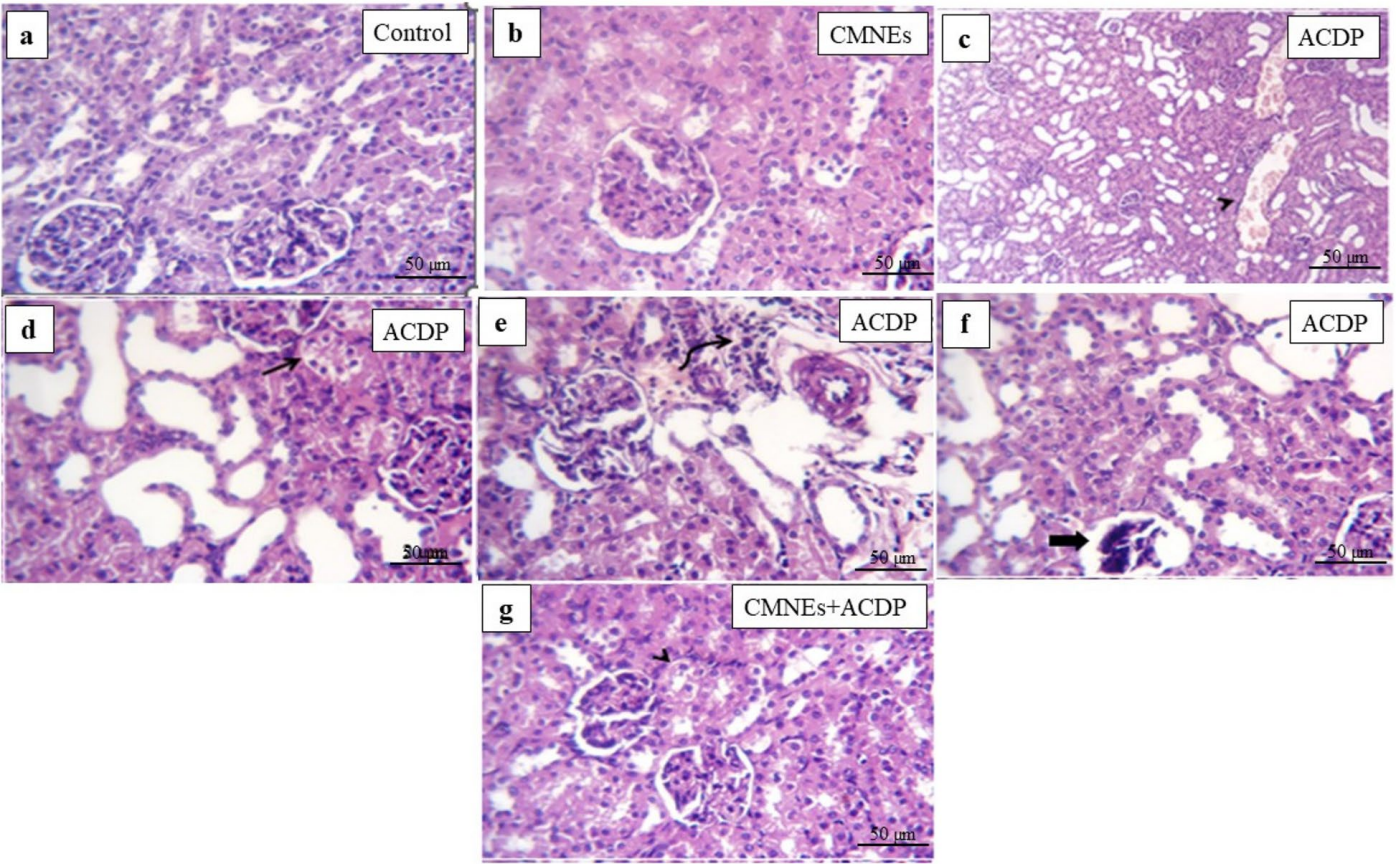
Photomicrograph of H&E-stained sections from kidney showing some parameters (Dilated tubular lumina, glomerular shrinkage, degenerative changes, necrotic changes, round cells infiltrations). **a** Normal glomerular architectures (arrow) and surrounding renal tubule (arrowhead) are in control. **b** normal architectures of the glomerular and surrounding tubules of the kidney. **c**, **d**, **e**, **f** Severe congestion of the renal blood vessels (arrowhead), dilatation of most tubules, perivascular round cells infiltration (curved arrow), Marked hydropic degeneration and necrosis of tubular epithelium (arrow) and atrophy of some glomerular tufts (thick arrow). **g** normal renal tubules and glomerular structures with some degenerative changes in the renal epithelium (arrowhead)

All the experimental groups underwent histopathological examination of the liver and kidney tissues. The central vein and hepatic parenchyma of the livers of both control and CMNEs-treated rats were histologically normal (Fig. 5a, b, Table S1). However, those of the ACPD-treated rats exhibited severe degenerated and necrotic hepatocytes with congested hepatic blood vessels (Fig. 5c, Table S1). The livers of the ACPD + CMNEs-treated rats demonstrated normal hepatic cords with degenerative changes within some hepatocytes (Fig. 5d, Table S1). The kidney tissues of the control and CMNEs-treated rats reflected normal architectures of the glomerular and surrounding tubules of the kidney (Fig. 6a, b, Table S1). ACPD-treated rats exhibited notable characteristics, entailing severe congestion of renal blood vessels (Fig. 6c, Table S1), dilatation of the majority of tubules (Fig. 6d, Table S1), perivascular infiltration of round cells (Fig. 6e, Table S1), and significant hydropic degeneration, necrosis of tubular epithelium, and atrophy of certain glomerular tufts (Fig. 6f, Table S1). In ACPD + CMNEs-treated rats, the kidney revealed normal renal tubules and glomerular structures with some degenerative changes in the renal epithelium (Fig. 6g, Table S1).

### Detection of the COX-2

**Fig. 7:**
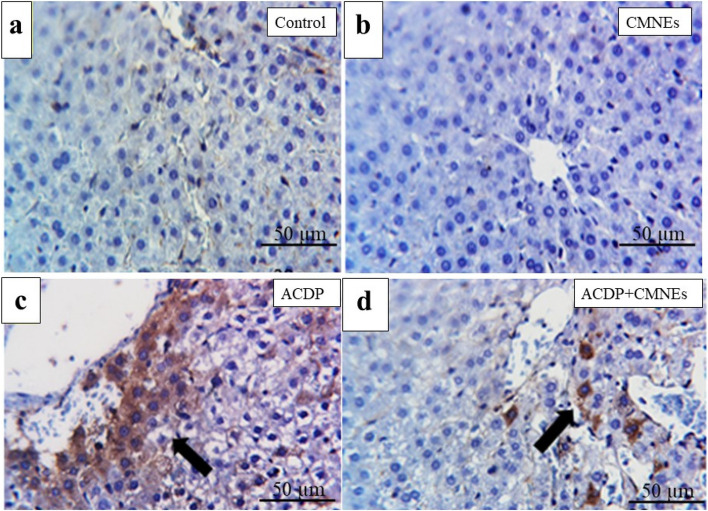
Photomicrographs of Cox2 immunohistochemistry staining in hepatic sections showing (**a**, **b**) negative expressions of Cox2 in control (GI) and CMNEs, respectively. **c** diffuse immunoexpression of Cox2 in ACDP-treated rats (arrow). **d** few numbers of immunostained cells by anti-Cox2 in ACDP + CMNEs treated rats (arrow). IHC counterstaining with Mayer’s hematoxylin. Scale bar 20 μm

**Fig. 8:**
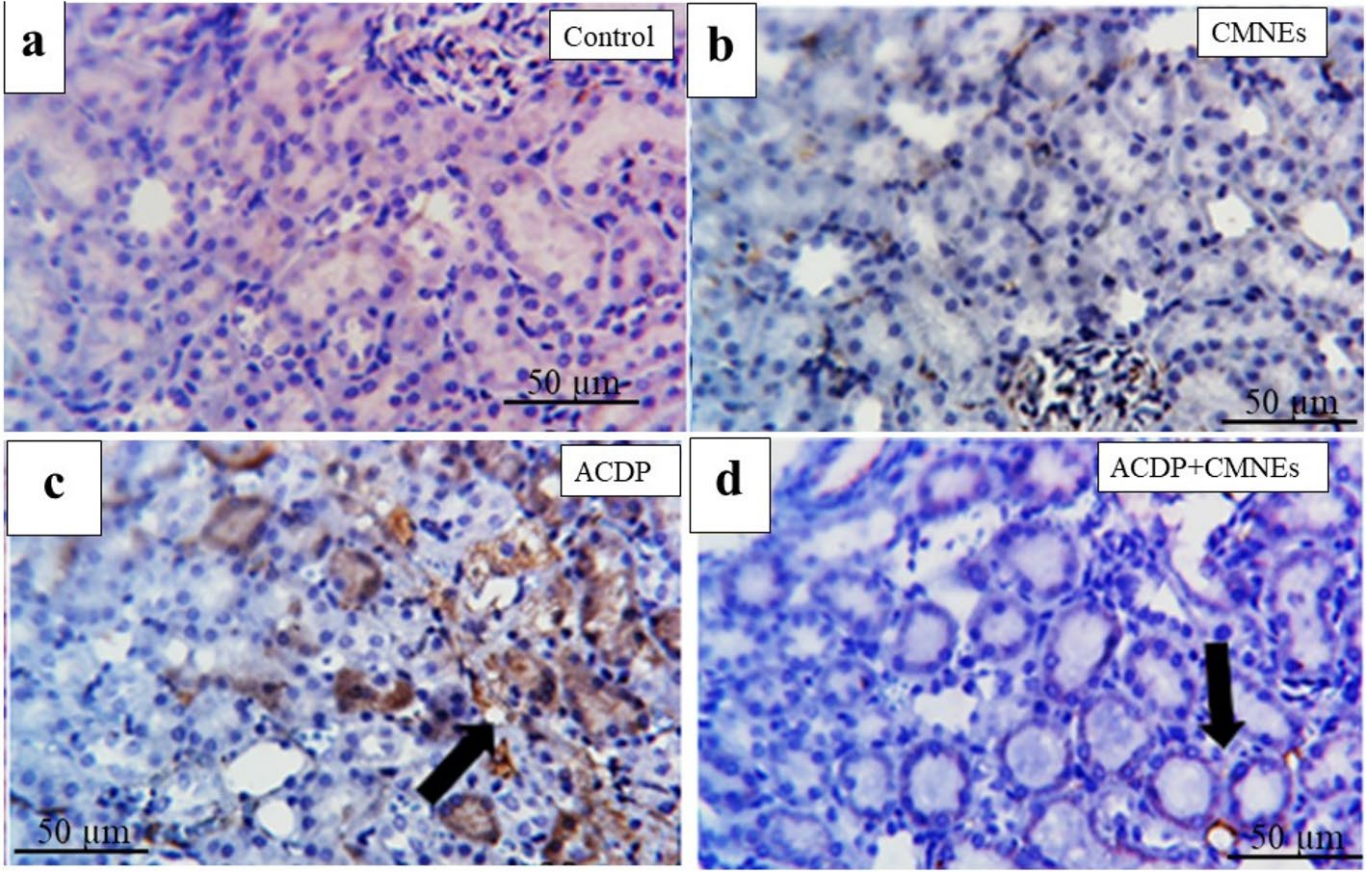
Photomicrographs of Cox2 immunohistochemistry staining in kidney showing (**a**, **b**) negative expressions of Cox2 in control and CMNEs, respectively. **c** large number of immunoexpressed cells with anti-Cox2 in ACDP-treated rats (arrow). **d** few numbers of immunostained cells by anti-Cox2 in ACDP + CMNEs treated rats (arrow). IHC counterstaining with Mayer’s hematoxylin. Scale bar 20 μm

**Fig. 9:**
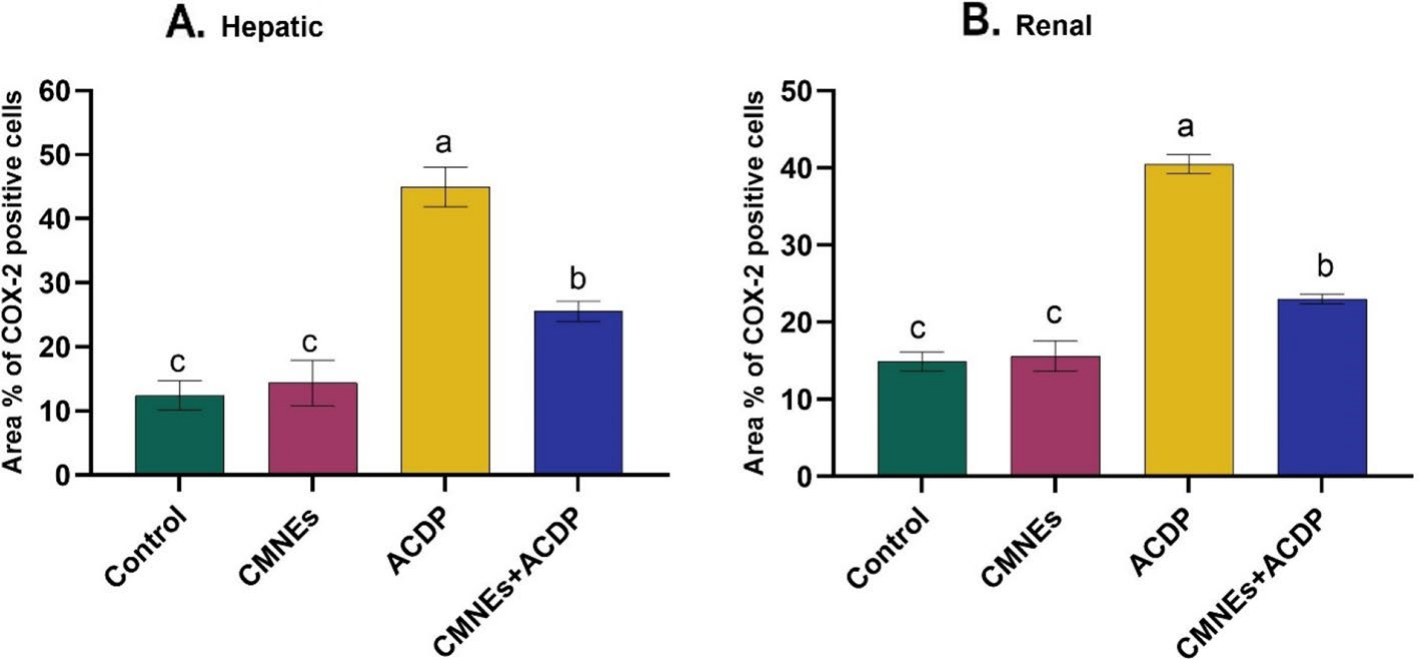
Area % of Cox-2 positive brown stained cells. In hepatic (**A**) and renal (**B**). All the values were expressed as mean *±* SEM. Different small letters indicate significance at *P* < 0.05

IHC was utilized to identify the location of COX-2 antigens in the rat liver and kidney tissues in all tested groups. Negative COX-2 staining was visible in the tissues of the control and CMNEs-treated rats (Figs. 7 and 8, b). In Fig. 6c, a diffuse immunoexpression of COX-2 antigen is depicted, unveiling widespread positive cytoplasmic staining (golden brown color) in the liver of ACPD-treated rats. Conversely, a few immunostained cells by anti-COX-2 in ACDP + CMNEs treated rats were delineated in Fig. 8d. In kidneys, large numbers of immunoexpressed cells with anti-COX-2 in ACDP-treated rats were illustrated as a diffuse positive cytoplasmic expression (golden brown) of COX-2 (Fig. 8c). Contrarily, the kidneys of rats given ACDP + CMNEs treatment displayed only a small number of anti-COX-2 immunostained cells (Fig. 8d). The % area of cox-2 was presented in Fig. 9.

### Molecular docking assay

**Fig. 10:**
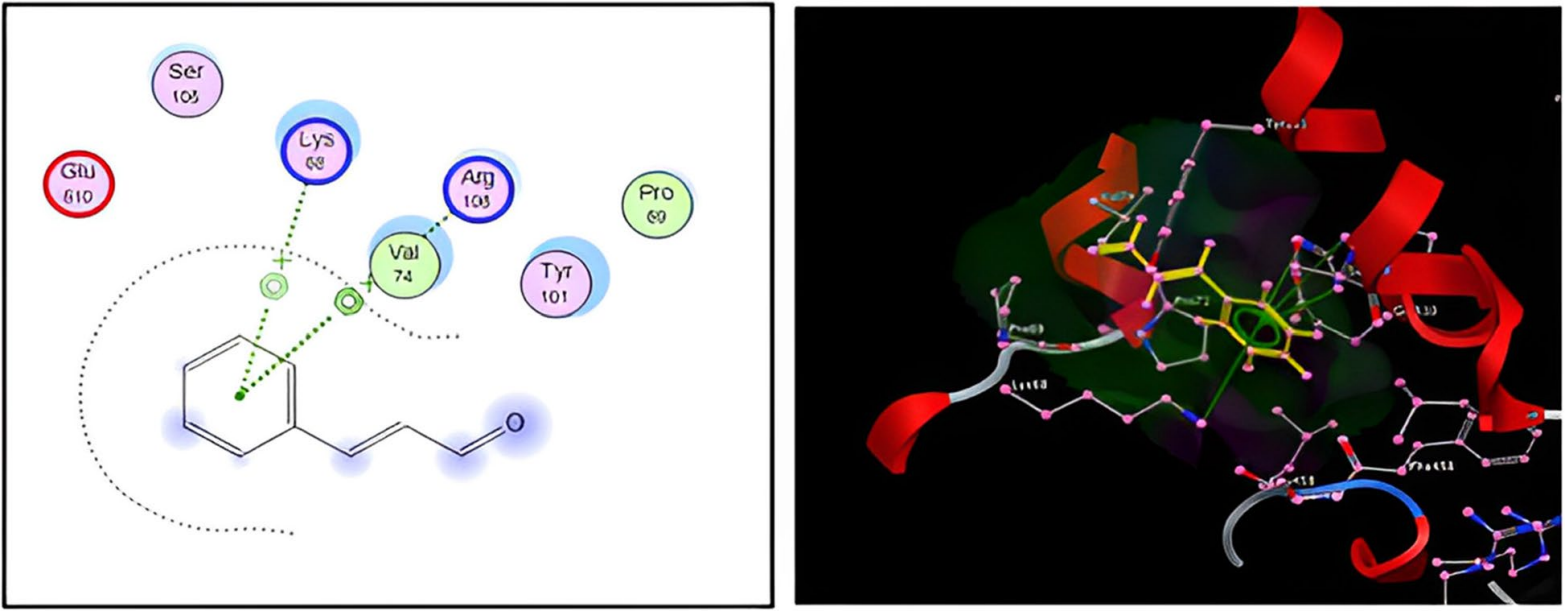
Docking view of binding interactions of major CMNEs (Cinnamaldehyde) constituent as a ligand with COX-2 as a receptor. Left: two-dimensional interaction diagram of constitute–receptor complexes. Right: the 3D complex structure and ligand bonds are depicted by yellow lines

By noticing the binding affinity between the main components of CMNEs (cinnamaldehyde) and COX-2, the molecular docking experiment validates the findings of immunohistochemistry, which unveil that CMNEs protect the liver and kidney tissues from the damaging effects of acetamiprid. The docking scores of interactions between cinnamaldehyde and COX-2 as ligands were delineated in Fig. 10. The docking analysis demonstrated that the investigated cinnamaldehyde had a low docking energy (-16.62 kcal/mol) and a high affinity for the active sites (target enzyme). Cinnamaldehyde as a ligand deeply enters COX-2’s hydrophobic pocket (1.01 Å) through two H-pi bonds with Arg 106 and Lys 68, surrounded by the residues Glu 510, Ser 105, Val 74, Tyr 101, and Pro 69.

## Discussion

Pesticides are persistent and shared in the environment, and they pose a risk to people and animals alike, damaging the ecosystem. They can also bioaccumulate and disrupt the food chain [48]. Nanoemulsions, which range in size from 20 to 500 nm, are sometimes called miniemulsions, submicron emulsions, or ultrafine emulsions [49]. There are three different kinds of nanoemulsions combining oil in water (O/W), and water in oil (W/O) [50]. Previous studies proved that nanoemulsion also exhibited anti-inflammatory and antidiabetic properties and potent biological effects against germs and fungi [26]. The *C. verum* EO-based NE exposed a conductivity value, pointing out the presence of slightly conductive ions, which could avoid the polarization of the electrode and boost its stability and nondeteriorating [51].

Moreover, due to the extreme negativity of the zeta potential (-24.2 ± 4.39), there was a significant level of stability [52]. The PDI value (0.24) indicated that the nanoemulsion had good physical stability because the Ostwald ripening was minimized [53], with low viscosity, which might be caused by the low oil content that delayed instability processes and produced oil droplets with a more uniform particle size [54]. Based on TEM evaluation, the droplets were spherical, and the particle size ranged from 22 to 37 nm, consistent with observations of dynamic light scattering. On the other hand, TEM was also employed due to the limitations of DLS’s capacity to analyze the nanoparticle structure in detail.

GC-MS is an excellent tool for studying the VOCs from spices and aromatic plants, even at trace levels [55]. Our results from the GC/MS study demonstrated that the extract was enhanced by various bioactive substances that could function as antioxidants, anti-inflammatory substances, and free radical scavengers [56]. Cinnamaldehyde is the highest concentration in the CMNEs. Our data is consistent with research by Wang et al. [56], reporting that cinnamon analysis produced cinnamaldehyde levels ranging from 0.023 to 0.29% w/w. Moreover, The main ingredient in cinnamon oil is cinnamaldehyde [57].

Furthermore, the concentration of cinnamaldehyde in cinnamon oil reached 12.01% [58]. These compositional changes could result from varied environmental (climatic, geographical, or seasonal) and genetic variations. In addition, The content of essential oils varies depending on several variables, including plant sections, the time of year, the extraction process, and the plants’ nutritional state [59].

Indefinite environmental pollutants have diverse detrimental health effects on people and animals, especially in poor nations [60]. ACDP-treated rats had significantly lower BWG than the control group. This can be interpreted as acetamiprid treatment, which indicates the potentially hazardous effects of this chemical [7]. Moreover, rats treated with ACDP at 30 mg/kg orally for 35 days had lower BWG [61]. In addition, a discernible decrease in the body weight of mice was marked in the ACDP-treated group compared to the control group [62]. This effect is likely due to the toxic impact of acetamiprid on physiological functions, which may include disruption of normal metabolic processes or organ function, leading to an overall decrease in the health and growth rate of the animals. The reduced body weight gain is a standard indicator of systemic toxicity in such exposure scenarios [63].

On the other hand, the body weight was recovered after the coadministration of CMNEs at 2 mg/kg BW. This result was congruent with the findings of Huang and Chen [64], who reported that CMNEs boosted the BWG of rats, possibly due to their regulatory effect in enhancing antioxidant activity. As a result, a disruption in food intake might be responsible for the decrease in body weight growth observed following ACDP administration. Our result was also supported by [64], who revealed that the cinnamon nanoemulsion was effective in lowering body weight loss in diabetic rats and showed that the effectiveness of the nanoemulsion absorption.

Hematological findings demonstrated a remarkable decline in RBC counts, Hb concentration, HC, and PLT levels in the ACDP-treated group relative to the control group. The lysis of RBC brought on by oxidative damage to the cell membranes generated by ROS might cause a decrease in both RBC and Hb [65]. Moreover, this decrease could be produced by a failure in the generation of red blood cells or by an increase in the destruction of erythrocytes. This might be why the anemia and decreased Hb levels are linked to these pesticides [66]. Our findings agreed with Singh et al. [66], who confirmed that male and female mice treated with ACDP had lower RBC counts and hemoglobin concentrations in their blood. Notably, higher WBC and lymphocyte counts following ACDP treatment mirrored an active immunological response in the animals, presumably due to pesticide-induced necrosis and tissue damage [67]. The rise in WBC counts may be a pathogenic reaction. These cells substantially impact during infestation by inducing the immune system and hemopoietic organs to create antibodies in response to the stress caused by ACDP [68]. The increase in WBC counts in rats receiving acetamiprid was verified by Celik et al. [69].

The investigation into oxidative stress markers reveals significant insights into the underlying mechanisms of acetamiprid-induced toxicity and the protective role of cinnamon nanoemulsion. Acetamiprid, a neonicotinoid pesticide, has been documented to induce oxidative stress by generating reactive oxygen species (ROS) in hepatic and renal tissues, leading to elevated levels of malondialdehyde (MDA), a primary marker of lipid peroxidation and cellular damage. This increase in MDA signifies enhanced lipid peroxidation, a direct consequence of oxidative stress and a critical factor in the pathogenesis of organ damage [70]. Furthermore, the overproduction of ROS in the liver precipitated by the ACDP metabolism by hepatic enzymes leads to oxidative damage such as lipid peroxidation, protein breakdown, and DNA damage [71]. Another study claimed that ACDP raised the level of MDA, signifying that lipid peroxidation had been induced. This could have resulted in the loss of membrane structure and function [61]. Antioxidant enzymes like SOD, CAT, and GPx protect cells from oxidative stress by converting superoxide radicals into less harmful substances like water and oxygen [72]. GPx safeguards cellular molecules by reducing hydroperoxides to water, while MDA, a lipid peroxidation product, indicates oxidative damage to cells [73]. In agreement with our research, ACDP-treated rats had significantly lower CAT, SOD, and GPx activities, as well as higher levels of MDA and H2O2. ACDP produces oxidative stress by generating hydroxyl radicals, superoxide anions, nitric oxide, and hydrogen peroxide, contributing to lipid peroxidation [74, 75]. In addition, Ghazanfari et al. [76] reported that the presence of oxidative stress in hepatic cells was suggested by enhanced lipid peroxidation, oxidation of thiol groups, and a decrease in SOD activity in hepatic tissues of ACDP-treated groups.

Conversely, cinnamon nanoemulsion, rich in antioxidant compounds such as phenolic acids and flavonoids, has demonstrated a potent capacity to scavenge these harmful ROS. Enzymatic antioxidants like superoxide dismutase (SOD), catalase (CAT), and glutathione peroxidase (GPx) play crucial roles in the body’s defense mechanism against oxidative stress. SOD catalyzes the dismutation of the superoxide anion into hydrogen peroxide, which is then further decomposed to water and oxygen by CAT and GPx, thus mitigating cellular oxidative damage [77]. Likewise, the level of CAT, SOD, and GPx activities decreased with ACDP-treated groups [78].

In contrast, CMNEs stopped the rise in MDA and H2O2 levels as well as the reductions in CAT, SOD, and GPx activity. Our results aligned with a recent study conducted by Berktas and Peker [27], exposing that coadministration of cinnamon along with malathion provoked higher CAT concentration compared to the administration of malathion alone. Additionally, the cotreatment of cinnamon with diclofenac sodium or cinnamon alone lessened the MDA level and enhanced the level of SOD and CAT compared with diclofenac sodium alone [79]. The study found that a high-dose nanoemulsion, rich in phenolic acids and flavonoids, significantly reduced MDA levels, indicating its potent antioxidant activity in decreasing oxidative damage [80]. The protective effects of cinnamon nanoemulsion in mitigating acetamiprid-induced toxicity can be attributed to its antioxidant properties, which enhance the activity of critical antioxidant enzymes and reduce markers of oxidative stress like MDA. These results provide a promising basis for further research into the use of plant extract nanoemulsions in protecting against chemical-induced organ damage [81].

ACDP may have both inflammatory and immunosuppressive effects [82]. This hypothesis is consistent with our observations that ACDP leads to detrimental biochemical impact on the liver, as seen by noticeably elevated blood levels of ALT, AST, and ALP, and that CMNEs inhibit these elevations. The damage in the liver of rats administrated with ACDP may be due to heightened permeability of the cell membrane [83]. Consequently, transaminases are released into the bloodstream, causing hepatocyte injury and numerous coagulation factors [84], and their inhibitors cannot be adequately synthesized, constricting blood arteries. These findings are consistent with those by Khovarnagh and Seyedalipour [14], reporting that ALT, AST, and ALP levels were elevated in serum after ACDP administration compared with control.

Moreover, the administration of cinnamon decoction remarkably curtailed AST, ALT, and ALP levels compared to restraint-stressed rats [85]. In addition, coadministration of cinnamon plus acetaminophen treatment significantly reduced AST, ALT, and ALP activities compared with acetaminophen alone [86]. Likewise, cotreatment with cinnamon oil plus deltamethrin group reduced the increased AST, ALT, and ALP activities [87].

The most noticeable histological variation showed up as diffuse patches of hydropic degeneration and necrotic hepatocyte evidence. On the contrary, in the kidney, severe congestion of the renal blood vessels, perivascular round cells infiltration, marked hydropic degeneration and necrosis of tubular epithelium, and atrophy of some glomerular tufts was recognized, which results in fluid leakage from the vascular compartment into the interstitium [88]. Our data aligns with research by Mondal et al. [89] that reported enlargement and petechial hemorrhages in the liver of rats exposed to ACDP. Moreover, ACDP may induce hepatocellular necrosis, fast hepatic architectural disorganization, sinusoidal structure disintegration, and blood pooling in the liver through these processes [90]. Furthermore, tubule deterioration and desquamation of the lining epithelium were discovered by Arıcan et al. [62] in ACDP-treated kidney rats. Additionally, in kidney rats treated with ACDP, Most tubules had coagulative necrosis in the lining epithelium and severe congestion in the cortical blood vessels [91]. Likewise, in ACDP-treated rats, tubular cells were wholly lysed, leaving the reticular framework, and there were degenerative and necrotic alterations in the kidney’s proximal and distal convoluted tubules [89].

Our findings proved that CMNEs may have had an ameliorative effect. All previously histological changes were ameliorated when CMNEs and ACDP were administered together, and all acetamiprid-induced toxic effects were modulated to within normal limits. Its antioxidant capabilities might be responsible for this protective function [87]. The investigation’s histopathological findings in the ACDP + CMNEs group improved, which was in line with the advancement of biochemical data. Compared to the ACDP group alone, there was a notable decline in the histological changes in this group, and the bulk of the liver and kidney tissue displayed essentially the same structure. Our results agreed with Kardan et al. [92], documenting that CMNEs might speed up healing due to escalated cellular infiltration in rats.

Moreover, CMNEs diminished the adverse effects of deltamethrin absorption and prevented its damaging immune system effects [87]. Furthermore, cinnamon ameliorated cypermethrin-induced histological alterations in the livers of rats [23]. Likewise, the authors in [93] argued that cinnamon protected the liver from the fatty alterations brought on by cholesterol in rats. An analogous investigation revealed that administering cinnamon extract ameliorated the histological alterations between rats given paracetamol [21]. Additionally, cinnamon has anti-inflammatory and antidiabetic properties and potent biological actions against germs and fungi. It has been used as an anti-inflammatory and anticancer medication. The activity is due to eugenol, trans-cinnamaldehyde, linalool, and other bioactive ‎components [94].

COX-2 is a crucial enzyme that has a role in the pathogenesis of hepatonephrotoxicity. It is commonly acknowledged that it can trigger inflammatory processes linked to many liver illnesses. Xanthine Oxidase speeds up the conversion of hypoxanthine to xanthine and xanthine to uric acid. H_2_O_2_ and ROS, which are byproducts of this process, play a significant role in the etiology of tissue damage [95]. Our assessments of biochemistry and histology were confirmed by the COX-2 IHC results, which did not expose any cytoplasmic expressions of COX-2 within hepatic and renal tubular cells in control and CMNEs-treated rats. In ACDP-treated rats, COX-2 was detected in the liver and kidney tissues to varied intensity, while immune expressions appeared in a few cells in the ACDP + CMNEs group. Our results are consistent with previous studies demonstrating that nuclear factor kappa B (NF-B) is involved in the upregulation of COX-2 [96]. In addition, the expression of COX-2 protein was intensified after exposure to DDD and DDE [97].

Moreover, it is hypothesized that COX-2 contributed to inflammation and tumor growth‎. Additionally, xenobiotics may precipitate COX-2 expression by activating NF-B [98]. Also, Expression of COX-2 is associated with inflammation and is released by an array of proinflammatory stimuli [99]. Interestingly, a diffuse positive cytoplasmic expression of COX-2 illustrates the enormous cytoplasmic immunoreactivity for detecting COX-2 antigen in the livers and kidneys of abamectin-treated rats [100].

Another intriguing finding supporting the immunohistochemistry results is that cinnamon aldehyde discloses a strong binding with the COX-2 enzyme, a key player in the pathogenesis of liver and kidney damage. This may be because cinnamaldehyde can operate as a free radical scavenger, antioxidant, and anti-inflammatory agent [101]. Attaining notable downregulation of COX-2 enzyme expression during oxidative stress could be a critical strategy in regulating liver and kidney destruction [100]. Moreover, trans-cinnamaldehyde treatment uncovered anti-inflammatory actions by downregulating COX-2 expressions compared with methotrexate alone [102]. Furthermore, cinnamaldehyde treatment significantly attenuated inflammation in mesenteric liver injuries by downregulating the expression of inflammation-related COX-2 [103].

Our hematological, biochemical, histological, IHC and molecular docking analysis manifested that CMNEs shielded the kidney and liver from renal-hepatic damage caused by ACDP because of their anti-inflammatory and antioxidant qualities.

## Conclusion

Our study underscores the significant risks posed by subchronic exposure to acetamiprid (ACDP), a common neonicotinoid insecticide increasingly detected as a dietary contaminant. The findings from our experimental model reveal that ACDP exposure can lead to substantial toxic effects on the hepatic and renal systems in rats, characterized by decreased activities of key antioxidant enzymes. Moreover, significant increases in liver enzymes and kidney markers injury—alongside marked histopathological damage to liver and kidney tissues highlight the severity of ACDP’s detrimental impacts. Importantly, our research demonstrates the therapeutic potential of cinnamon nanoemulsions (CMNEs) in mitigating these adverse effects. The coadministration of CMNEs with ACDP substantially ameliorates the observed oxidative stress and inflammation in the hepatic and renal systems. This protective effect is likely mediated through the upregulation of antioxidant defenses, as evidenced by normalized oxidative stress markers and improved histological outcomes. The molecular interactions between cinnamaldehyde, a key component of CMNEs, and cyclooxygenase 2 (COX-2), further elucidate the mechanistic basis of CMNEs’ protective efficacy. These findings reinforce the need for caution in the environmental and dietary exposure to acetamiprid and highlight the promising role of bioactive nanoparticle formulations from medicinal plants in enhancing antioxidant defenses. Applying such nanoparticles could serve as a viable strategy to counteract the oxidative stress and organ toxicity induced by various environmental contaminants. Future research should aim to explore the broader applicational scope of these nanoparticles across different models and setups to harness their therapeutic potential fully.

### Supplementary Information

Supplementary Material 1.

## Data Availability

No datasets were generated or analysed during the current study.
