# Supplementary material for: Cinnamon nanoemulsion mitigates acetamiprid-induced hepatic and renal toxicity in rats: biochemical, histopathological, immunohistochemical, and molecular docking analysis
Source: BMC Vet Res. 2024 Jun 12;20:256. doi: 10.1186/s12917-024-04084-x (PMC11167909; doi:10.1186/s12917-024-04084-x)
Supplement: Supplementary file 1 — Supplementary Material 1. [file 12917_2024_4084_MOESM1_ESM.docx]

Table S1: The represented scores in the table are the mean lesion scores. Histologic lesions were scored for severity (-= No altrerations, += Mild altrerations , ++= Moderate altrerations, +++= Severe altrerations).

| organ | Main lesions | Control | Cinnamon | acetamiprid | acetamiprid + Cinnamon |
| --- | --- | --- | --- | --- | --- |
| Liver | Degenerative changes | - | - | +++ | + |
|  | Necrotic changes | - | - | +++ | - |
|  | Congested blood vessels | - | - | + | - |
|  | Round cells infiltration | - | - | + | + |
| Kidney | Dilated tubular lumina | - | - | ++ | - |
|  | Glomerular shrinkage | - | - | ++ | - |
|  | Degenerative changes | - | - | +++ | + |
|  | Necrotic changes | - | - | + | - |
|  | round cells infiltrations | - | - | + | + |

**The methodology for assessing antioxidant markers and oxidative stress markers involves specific techniques and assays to measure the activity of enzymes involved in combating oxidative stress and damage. Here's a detailed description of each method:**

1. **Superoxide Dismutase (SOD) Activity Measurement:**

SOD activity was measured using a kit from Biodiagnostic, Cairo, Egypt, following the technique described by Nishikimi et al. [40]. The assay typically involves the inhibition of the autoxidation of adrenaline to adrenochrome by superoxide radicals generated by a xanthine-xanthine oxidase system. The decrease in absorbance at 440 nm is monitored spectrophotometrically over time, reflecting the scavenging of superoxide radicals by SOD. SOD activity is expressed in units per milligram of protein

1. **Catalase (CAT) Activity Measurement:**

CAT activity was measured according to the method outlined by Aebi [41], utilizing a kit from Biodiagnostic, Cairo, Egypt.The assay involves the decomposition of hydrogen peroxide (H2O2) by catalase enzyme present in the sample.The decrease in absorbance at 510 nm is monitored spectrophotometrically as H_2_O_2_ is converted to water and oxygen.

1. **Glutathione Peroxidase (GPx) Activity Measurement:**

GPx activity was evaluated using spectrophotometry based on the method described by Paglia and Valentine [42]. The assay involves the oxidation of glutathione (GSH) by GPx in the presence of hydrogen peroxide (H_2_O_2_) and glutathione reductase. The reduction of oxidized glutathione (GSSG) back to GSH by glutathione reductase consumes NADPH, resulting in a decrease in absorbance at 340 nm.GPx activity is expressed in units per milligram of protein, indicating the amount of enzyme required to catalyze the oxidation of substrates per unit time.

1. **Malondialdehyde (MDA) Measurement:**

MDA, a marker of lipid peroxidation and oxidative stress, was quantified using a kit from Biodiagnostic, Cairo, Egypt, as per the method developed by Ohkawa et al. [43]. The assay involves the reaction of MDA with thiobarbituric acid (TBA) under high temperature and acidic conditions, forming a colored complex. The absorbance of the colored complex is measured spectrophotometrically at 532 nm. MDA concentration is expressed in mmol per milligram of protein, indicating the amount of MDA produced per unit protein content.

**Hydrogen peroxide (H_2_O_2_) levels** were measured at 610 nm using the methodology proposed by Pick and Keisari [44], which likely entails an enzymatic assay involving the reaction of H_2_O_2_ with a peroxidase enzyme and a chromogenic substrate. A kit acquired from Biodiagnostic in Cairo, Egypt, was utilized, providing standardized reagents and instructions for conducting the assay. Biological samples containing H_2_O_2_ were treated to release the molecule if bound or in a complex form, followed by the addition of a reagent mixture containing peroxidase and a chromogenic substrate. After allowing the reaction to proceed, the absorbance of the colored product was measured spectrophotometrically at 610 nm, and the H_2_O_2_ concentration was determined by comparing the absorbance readings to a standard curve prepared using known concentrations of H_2_O_2_. This method enables the quantitative assessment of H_2_O_2_ levels in biological samples, offering insights into oxidative stress and redox signaling mechanisms.
